# Neural Network for Metal Detection Based on Magnetic Impedance Sensor

**DOI:** 10.3390/s21134456

**Published:** 2021-06-29

**Authors:** Sungjae Ha, Dongwoo Lee, Hoijun Kim, Soonchul Kwon, EungJo Kim, Junho Yang, Seunghyun Lee

**Affiliations:** 1 Spatial Computing Convergence Center, Kwangwoon University, 20 Kwangwoon-ro, Nowon-gu, Seoul 01897, Korea; sungjae@kw.ac.kr (S.H.); ejkim1@kw.ac.kr (E.K.); 2 Department of Plasma Bio Display, Kwangwoon University, 20 Kwangwoon-ro, Nowon-gu, Seoul 01897, Korea; led0121@kw.ac.kr (D.L.); hoi97@kw.ac.kr (H.K.); 3 Department of Smart Convergence, Kwangwoon University, 20 Kwangwoon-ro, Nowon-gu, Seoul 01897, Korea; ksc0226@kw.ac.kr; 4 Agency For Defense Development, P.O. Box 132, Songpa-gu, Seoul 05661, Korea; yangstar999@add.re.kr; 5 Ingenium College of Liberal Arts, Kwangwoon University, 20 Kwangwoon-ro, Nowon-gu, Seoul 01897, Korea

**Keywords:** convolutional neural network, deep learning, magnetic impedance, metal detection, recurrent neural network, sensor, signal processing

## Abstract

The efficiency of the metal detection method using deep learning with data obtained from multiple magnetic impedance (MI) sensors was investigated. The MI sensor is a passive sensor that detects metal objects and magnetic field changes. However, when detecting a metal object, the amount of change in the magnetic field caused by the metal is small and unstable with noise. Consequently, there is a limit to the detectable distance. To effectively detect and analyze this distance, a method using deep learning was applied. The detection performances of a convolutional neural network (CNN) and a recurrent neural network (RNN) were compared from the data extracted from a self-impedance sensor. The RNN model showed better performance than the CNN model. However, in the shallow stage, the CNN model was superior compared to the RNN model. The performance of a deep-learning-based (DLB) metal detection network using multiple MI sensors was compared and analyzed. The network was detected using long short-term memory and CNN. The performance was compared according to the number of layers and the size of the metal sheet. The results are expected to contribute to sensor-based DLB detection technology.

## 1. Introduction

Recently, deep learning [1,2] has been proven effective and successful in many fields in science and engineering such as medical diagnoses, image [3,4,5,6], signal [7,8] and speech recognition, financial services, autopilot in automotive scenarios, and many other engineering and medical applications. In signal processing, most of the data are obtained in units of time columns. Accordingly, deep learning is being used in fields such as sensors that measure continuous values or periodic values. In the case of periodic signals, deep learning determines the correlation between the previous data and existing data, or it learns the continuity of data in time-series data, thereby providing high detection and prediction rates.

The magnetic impedance (MI) effect [9] is a phenomenon in which the impedance of a magnetic object changes according to the strength of an external magnetic field. This electromagnetic phenomenon [10] occurs when a pulsed current or high-frequency current that causes a skin effect is applied to a magnetic object. By applying the MI effect, an improved result is produced when an amorphous wire [11,12] is used as a sensor material. Modern sensors apply a pulsed magnetic field of 0.5 to 1 GHz to achieve high sensitivity. Depending on its strength, the pulsed magnetic field can be employed in various applications that use sensors, such as geomagnetic measurement, drone control, electronic mapping, foreign object detection, and autonomous driving. Moreover, it can be applied as a metal detector in foreign matter detection. In general, it is also used in mine detection, metal separation, and security checkpoints at airports.

In the past, deep learning has been applied in the field of signal processing, where it is mainly used for periodic signals. Data from MI sensors that respond to changes in the magnetic field are highly anomalous and varied. In this study, the detection performances of the data obtained from an MI sensor according to a signal-processing-based filtering method and a deep learning-based model were compared. Based on the data anomalies of the MI sensor, the detection performances using the respective image processing convolutional neural network (CNN) model and the recurrent neural network (RNN) model, which are widely employed for signal processing, were compared.

## 2. Related Works

### 2.1. Magnetic Sensor

**Figure 1:**
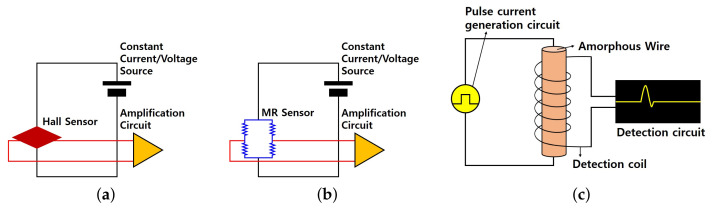
Representative magnetic sensors. (**a**) is the hall sensor and (**b**) is the MR sensor, (**c**) is the MI sensor.

Magnetic sensors measure the size and direction of a magnetic field. They are divided into different types according to their purpose. Some examples include Hall [13], magnetic resonance [14], and MI [15] sensors. Hall sensors use the Hall effect to measure magnetic flux density. The voltage is output in proportion to the magnetic flux density. It is mainly used for doors or laptops. The MR sensor measures the magnitude of a disturbance by utilizing the change in the electrical resistance of an object according to a magnetic field. Compared to the Hall sensor, it consumes less power and has higher sensitivity. It is used for electronic compasses and for motor rotation and position estimation. The MI sensor employs a special amorphous wire and applies the MI effect. It is more than 10,000 times more sensitive than the Hall sensor, and it can measure even minute changes in geomagnetism. The sensor power consumption includes ultra-low current consumption and involves methods such as magnetic induction. Moreover, power consumption can be applied to detect biomagnetic fields via human magnetic electrocardiogram (ECG), human white ECG, and human magnetic ECG at room temperature [16,17]. Figure 1 shows each sensor.

### 2.2. Convolutional Neural Networks

**Figure 2:**
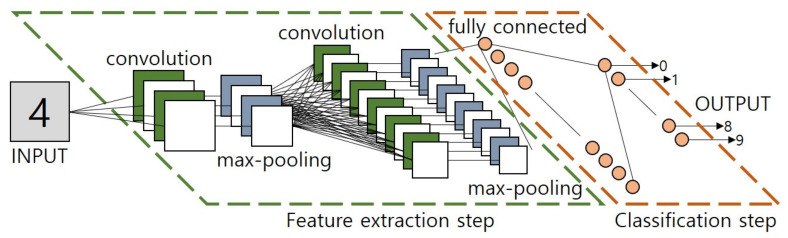
LeNet architecture.

CNN [18,19,20] is a type of artificial neural network. It is one of the most widely used algorithms in recent years in the field of image processing based on deep learning. It was developed to effectively process images by applying convolutional operations. This method is largely divided into a part that extracts features of an image and a part that classifies it. The feature extraction region is composed of several convolution and pooling layers. The classification is a process of classifying the extracted feature values by adding a fully connected layer [21,22,23,24]. A representative method with such a structure is LeNet, which classifies MNIST dataset, a handwritten data set. The structure is shown in Figure 2.

### 2.3. Recurrent Neural Networks

**Figure 3:**
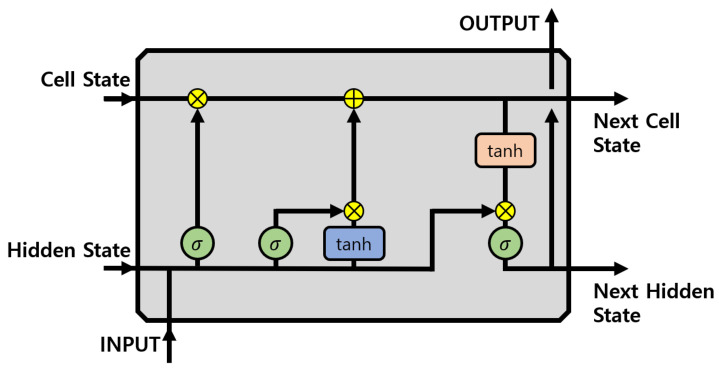
LSTM block diagram.

RNN [25] is a technique that performs classification or prediction by learning sequential data by circulating the output of the hidden layer back to the input. Unlike the existing deep neural network (DNN) [26], the RNN is a structure in which parameters are shared and cycled for each layer. This structure allows data from the past to affect those of the present and future. This makes it possible to classify or predict data. However, since the RNN receives only the output value of the previous step, status information is insufficient. To solve this problem, long short-term memory (LSTM) [27] has been proposed. LSTM is a method of delivering status information together with the output of the previous step. It thereby solves the problem of loss in the previous step. Figure 3 is a LSTM block diagram.

## 3. Proposed Method

**Figure 4:**
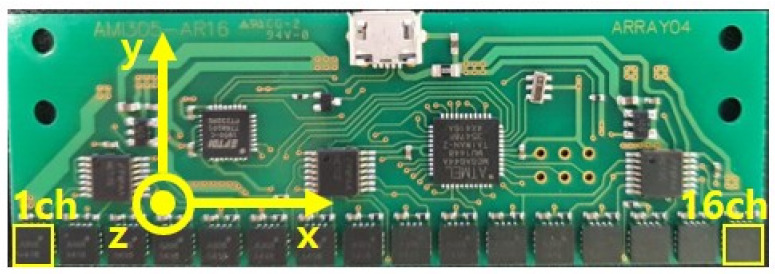
16ch MI sensor.

In this study, an AICHI AMI305-AR16 sensor with 16 MI sensors was used. In the raw data extracted from this sensor, the CNN and RNN methods for detecting metal objects were compared and analyzed. In general, for temporal data such as those obtained by sensors, the RNN deep learning method has shown good performance. However, the data measured by several identical sensors are composed of an array of data such as images. These data can be expected to be detectable through a CNN. Figure 4 shows the AICHI AMI305-AR16 sensor used in the experiment. It has a total of 16 sensors. The each sensor acquires 1 raw data per 8 msec. The experiment was conducted mainly on the z-axis. The x, y, and z axes were used for training and testing.

### 3.1. Data Collection

**Figure 5:**
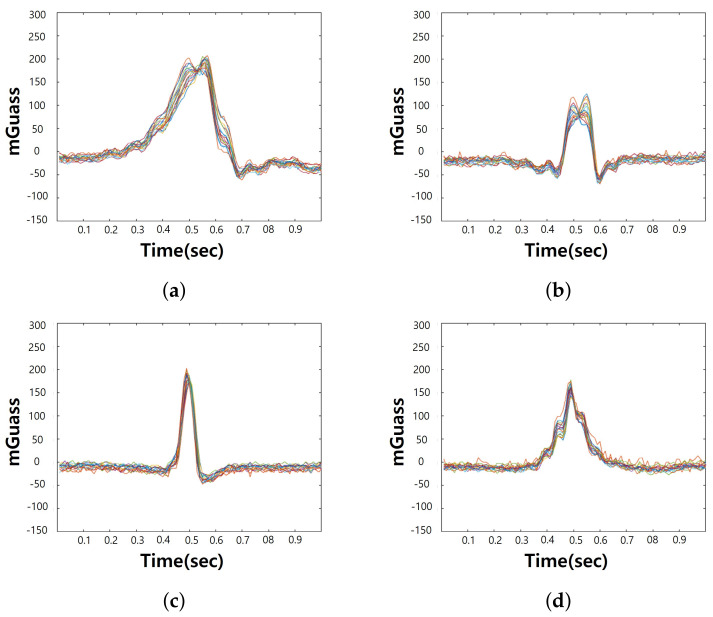
Data samples of z-axis. (**a**) is z-axis of 16ch sample data 1 and (**b**) is z-axis of 16ch sample data 2, (**c**) is z-axis of 16ch sample data 3, (**d**) is z-axis of 16ch sample data 4.

In this study, a square metal plate (2T) was used to construct a data set for use in deep learning model training and verification. The size was 30×30, 50×50, and 70×70, respectively, and the unit was centimeters. When performing the measurement, the distance between the sensor and the metal plate was 30 cm, 40 cm, and 50 cm, respectively, for each metal plate. We maintained fairness by experimenting at a constant speed. Measurements were made at a constant speed using an electric motor. The object speed of 2 m/s was used to build the data. Figure 5 shows the raw data of samples measured while moving the metal plate at 2 m/s when the distance between the 30×30 (cm) metal plate and the sensor was 15 cm. The z-axis graph is the part where the metal was detected. When constructing the experiment environment, the metal plate was moved in the direction of the z-axis of the sensor, and an example image was visualized with data on the z-axis.

**Figure 6:**
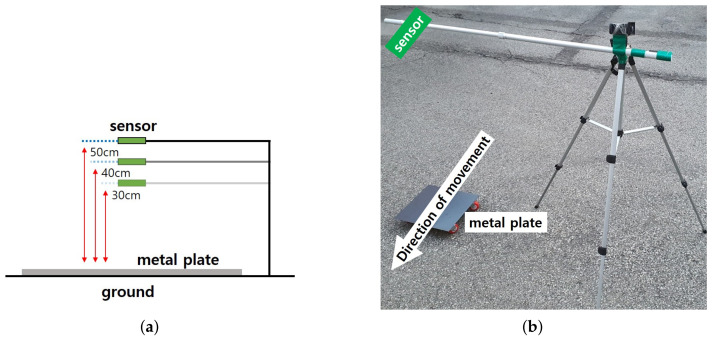
Experimental environment. (**a**) is experimental design and (**b**) is external experimental environment.

Figure 6 shows the equipment used and the experimental environment for the data measurement and construction. Wheels made of aluminum and rubber were installed on the metal plate to move the metal plate at 2 m/s. Aluminum and rubber are materials that do not respond to the MI sensor and do not affect data measurement. In addition, the distance between the metal plate and the sensor was set to 30 cm, 40 cm, and 50 cm, respectively. Accordingly, the experiment was conducted by changing the height of the sensor. The intensity of the sensor was not detected after 60 cm, so it was measured between 30 and 50 cm. As it moves in the direction of the arrow in the figure, the sensor z-axis data change was measured to be large.

### 3.2. Convolution Neural Networks-Based Signal Learning

**Figure 7:**
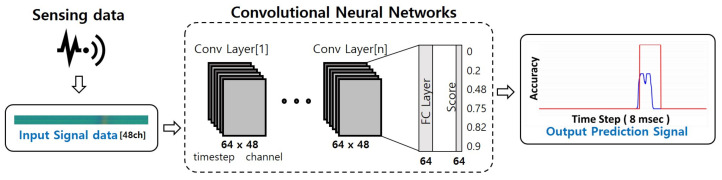
Signal learning with convolution neural networks architecture.

Introduced in Section 2.2, the CNN effectively processes array data such as images. It is advantageous for extracting similar features from images, and it can extract non-contiguous data. The measured value of the MI sensor may not be continuous. If the measurement distance increases, the continuity and repeatability of the data become ambiguous, and the detection rate in the RNN may decrease. Here, these data were imaged and the detection results using the CNN were analyzed. The results of the CNN model shown in Figure 7, which is composed of a simple shallow structure, were used in a comparison with the RNN model results.

**Figure 8:**
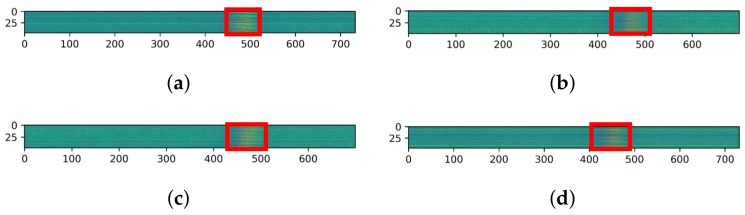
CNN input data samples. (**a**) is CNN sample array data 1 and (**b**) is CNN sample array data 2, (**c**) is CNN array image data 3, (**d**) is CNN array image data 4.

The CNN model input is received as a 2–3D array. However, the data consists of time-ordered signals and must be converted into 2–3D arrays of constant size. Therefore, to use the raw signal as CNN input, we rearranged the values of the x, y and z axes extracted from the 16 channel into a timeseries× 48. 48 high data array created. Figure 8 is an example of a data array.

In Figure 8, the area where the signal from the sensor reacts—owing to the movement of a metal object—appears in red and yellow. When performing the image labeling work, the corresponding area was treated as the correct answer.

### 3.3. Recurrent Neural Networks-Based Signal Learning

**Figure 9:**
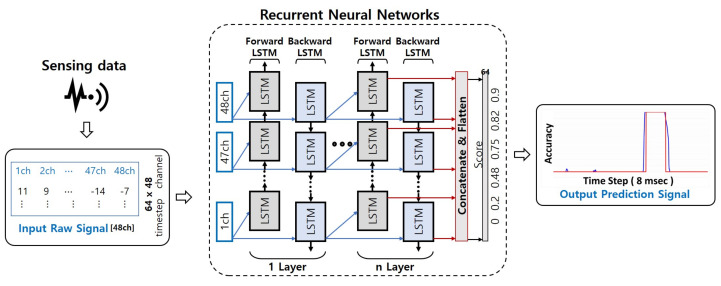
Signal learning with convolution neural networks architecture.

Introduced in Section 2.3, the RNN effectively processes temporal data measured by a sensor. Unlike the CNN model, it shows excellent performance in analyzing continuous and repetitive data. If the measurement distance is close, since the data are regular and continuous, the RNN detection performance may be high. The results of the RNN model used this study were applied in a comparison with those of the CNN model. Similar to the CNN model, it was composed of a shallow structure and a LSTM layer, which is often used in RNNs. To compare and analyze the results for each layer, each of the odd-numbered layers was configured. Figure 9 shows the structure of the RNN model.

**Figure 10:**
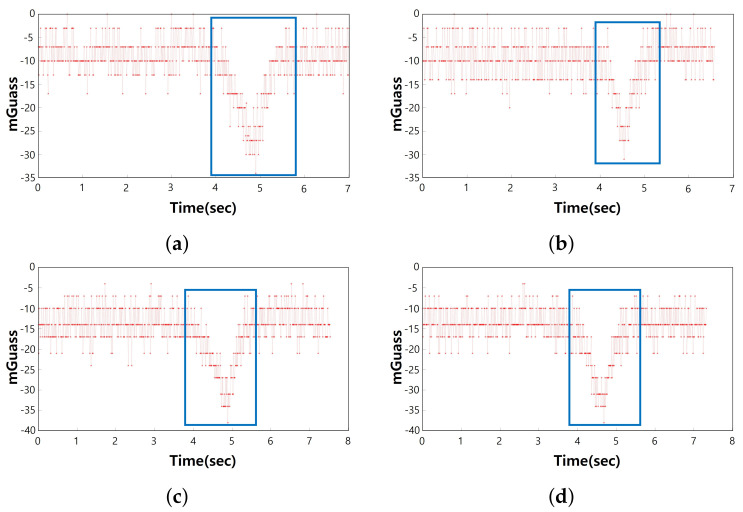
Raw dataset 30 cm × 30 cm × 30 cm samples. (**a**) is z-axis 1ch of sample 1 data and (**b**) is z-axis 1ch of sample 2 data, (**c**) is z-axis 1ch of sample 3 data, (**d**) is z-axis 1ch of sample 4 data.

The raw signal was used in the same way as it was for the input of the RNN model. The raw signal value was based on the time sequence and was contained in a csv file. Each of the 16 channels had 48 values per time column resulting from the extraction of the values of the x, y, and z axes. Figure 10 is an example image of graphing the time-series data. The graphing was performed on the z-axis of one channel. The raw signal was used in the same way as it was for the RNN model input. The raw signal value was based on the time sequence and was contained in a csv file. Each of the 16 channels had 48 values per time column resulting from the extraction of the values of the x, y, and z axes.

In Figure 10, the red box is the time when the sensor reacted as the metal object moved. The area was treated as the correct answer when labeling the data.

### 3.4. Network Implementation

Since this study was intended to detect metal objects using sensors, learning and experiments were conducted using TensorFlow 2.4 based on the CPU deep learning library. Learning and experimentation were conducted with AMD Ryzen 4500U and 8 GB of RAM. The CNN was composed of a convolution layer and a fully connected layer, and the RNN was composed of a LSTM network. ReLU [28] was used as the activation function for the CNN network, and Sigmoid [29,30] was employed as the activation function for the RNN network.

A network comparison experiment was conducted by connecting layers 1, 3, 5, 7, and 9 in series to compare the amount of required computation and the accuracy according to each layer. In addition, the performance of each network was compared and analyzed according to the distance from the metal object to the sensor.

Equation (1) was applied as the L1 loss function (mean absolute error) as the loss function used for CNN and RNN training in this study.

(1) $$L_{1}=\sum_{i=1}^{n}\left|y_{i}-f\left(x_{i}\right)\right|$$

Here, *y* is the correct answer of the data, and f(x) is the prediction result of the deep learning network. *x* is the input time sequence data. Accordingly, the difference between the predicted value of the network and the correct answer was learned. At this time, a batch was created and calculated to have popularity by simultaneously calculating several values. Sixty-four batches and 128 batches were composed. The Adam optimizer [31] was used as the neural network optimization method.

## 4. Experimental Results

### 4.1. Evaluation Index and Parameters

The data were constructed ten times for each distance (30 cm, 40 cm, 50 cm) and size of a metal object (30 cm × 30 cm, 40 cm × 40 cm, 50 cm × 50 cm) from the sensor to organize the learning data and test data. The ratio of the training data and the experimental data was 8:2 when the experiment was conducted. When evaluating each model through the experimental data, the accuracy of the prediction result was measured using Equation (2).

(2) $$accuracy=\frac{\sum_{i=0}^{m}\left(y-f\left(x_{l}\right)\right)}{m}\times 100$$

where *m* is the total number of time sequence data, *y* is the correct answer data, and $x_{l}$ is the *l*-th data.

**Figure 11:**
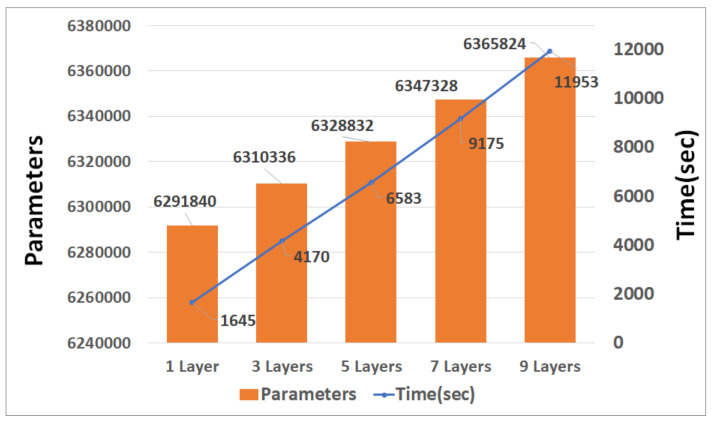
Graph of CNN parameters and training times.

**Figure 12:**
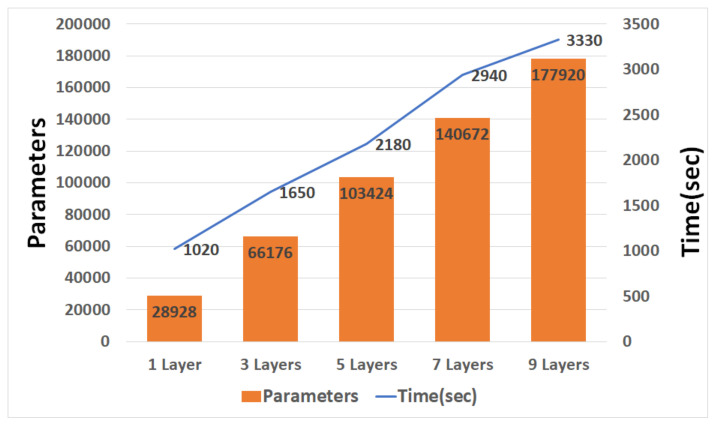
Graph of LSTM parameters and training times.

**Figure 13:**
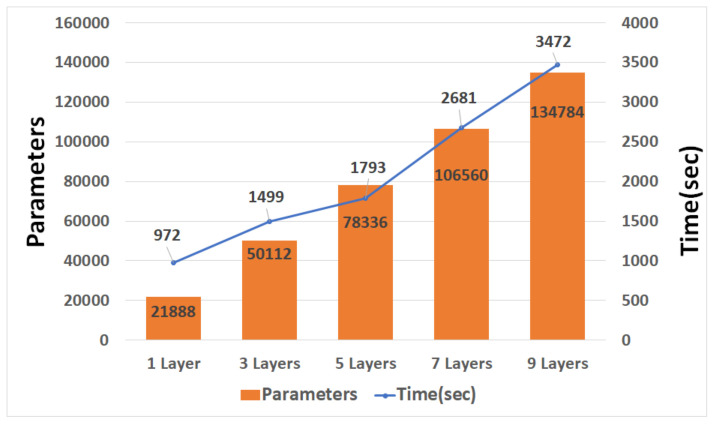
Graph of GRU parameters and training times.

Figure 11, Figure 12 and Figure 13 are graphs of the parameters and learning times according to CNN, and GRU depth. The parameter increases according to the depth and the time required for learning increases accordingly. Unlike RNN, CNN is a fully connected layer with most of its parameters; thus, there are few parameter changes due to the addition of convolution. However, it has more parameters than LSTM and GRU, and this affects the learning time.

When the CNN and LSTM each had nine layers, the learning time difference was approximately fourfold. As the number of layers decreased, the difference in learning time decreased.

### 4.2. CNN and RNN Results Comparision

**Figure 14:**
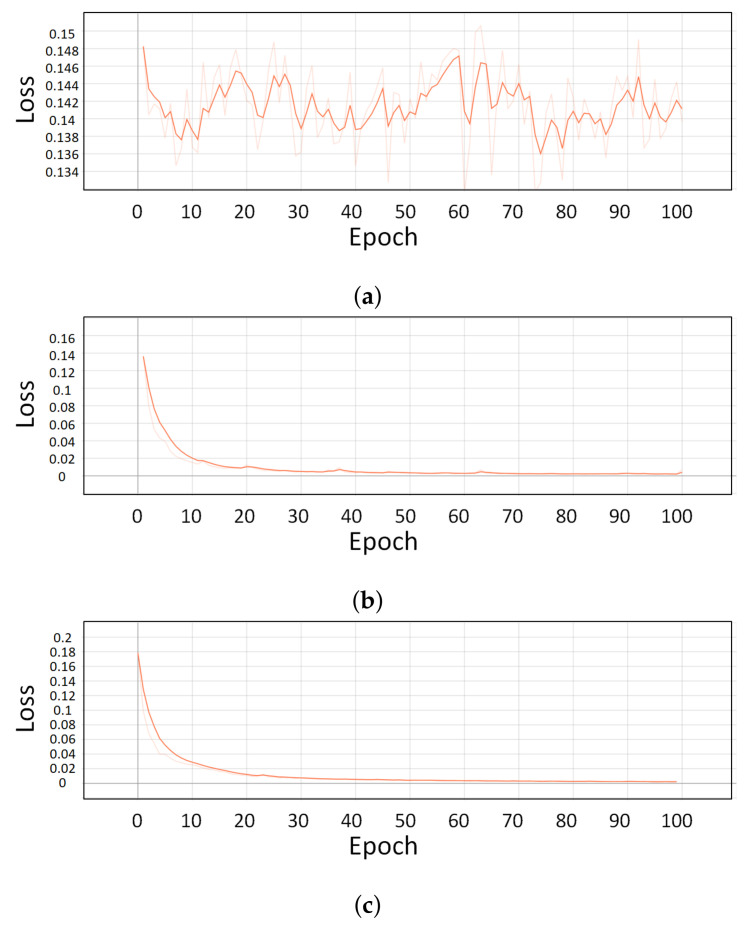
Graphs of the loss function. (**a**) is Graphs of the CNN loss function, (**b**) is Graphs of the LSTM loss function and (**c**) is Graphs of the GRU loss function

Figure 14 are graphs of the loss function. In the case of RNN-based LSTM and GRU, the loss value is sequentially decreasing. However, in the case of CNN, the loss value does not decrease and it shows an unstable shape in learning. This is because the data type received from the sensor is inappropriate in the CNN training process.

Table 1, Table 2 and Table 3 are performance comparisons for each layer depth of the CNN, LSTM and GRU. The performance of each layer was compared according to the distance between the steel plate and the sensor.

In general, both the CNN, LSTM and GRU showed improved performance as the network deepened. However, depending on the type and amount of data, different results may be displayed. In the case of the CNN, when the distance between the metal object and the sensor was significant, the deep network structure demonstrated high performance. This means that, when the learning data was ambiguous and implied semantic data, the semantic features were extracted through a deep network. In the case of a shallow network, features were extracted similarly to the input data, and the performance deteriorated when the input data was ambiguous. Therefore, the CNN showed high performance as a shallow network when the sensor was close to a metal object, and high performance as a deep network when the distance increased.

In the case of the LSTM, the layer depth did not affect the distance between the sensor and the metal object, and the results were consistent. Layers 1 and 3, which were shallow networks, did not properly learn the interrelationships of the time-series data, resulting in poor performance. In the case of layers 7 and 9, which were deep networks, it was advantageous when learning a large amount of data. Moreover, there was difficulty in learning because the amount of data in this study was small. The LSTM showed the highest performance at the fifth layer.

In the case of GRU, the depth of the layer has no effect on the distance between the sensor and the metal object. It showed relatively poor results in shallow networks such as LSTM, and showed the best results in the 7 layer.

The RNN-based showed better performance than the CNN-based because the RNN-based performs learning, including of the interrelationship of time-sequence signals. The CNN-based is a method of extracting features from an image, and performance can be improved if time-sequence data show a certain change. However, as in this study, it was difficult to learn when the signal change was ambiguous when the sensor responded to the training data and the experimental data.

**Figure 15:**
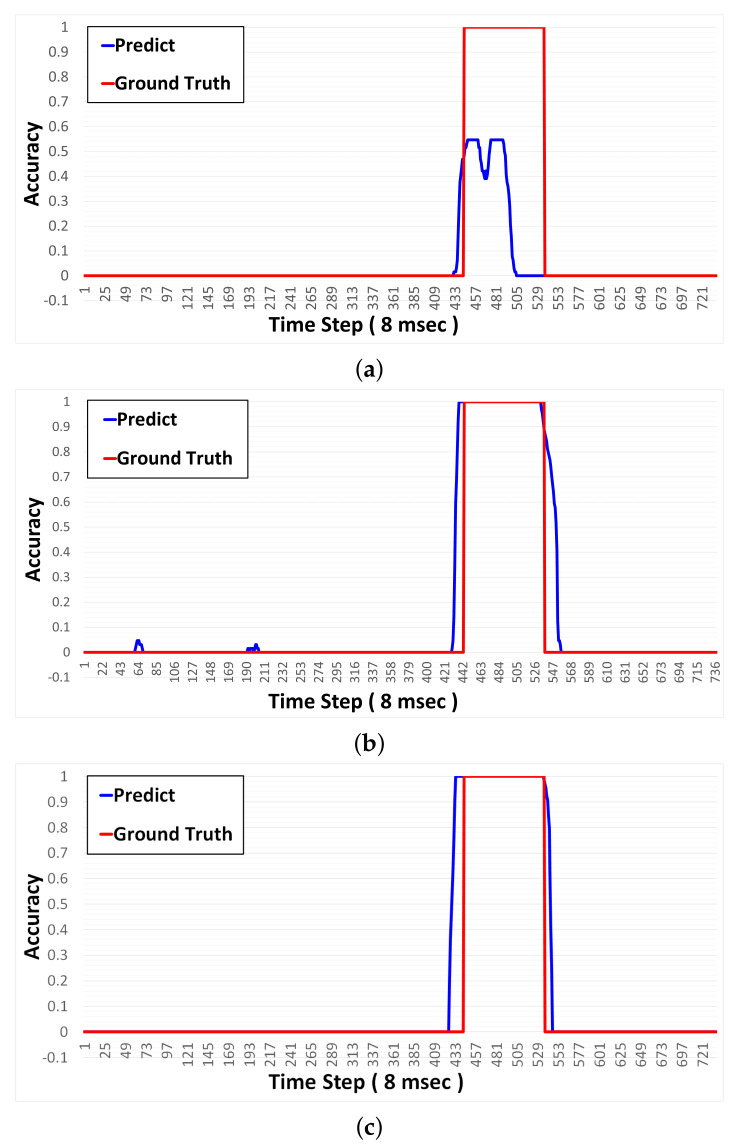
Network prediction results example. (**a**) is CNN prediction, (**b**) is RNN prediction and (**c**) is GRU prediction.

Figure 15 is a graph image of the raw data, the correct answer of the data, and the predicted result through the network. The blue line in (a) is the raw data measured from the sensor, and the orange line is the correct answer labeling of the data. The blue line in (b) represents the probability of the presence of the metal predicted by the learned network, and the orange line represents the correct answer area with the metal. The number “1” denotes the area in which the sensor reacts to the presence of metal, and “0” indicates the area in the static state when there is no metal. Only when the network learned in Figure 15 detects a metal object with 80% or higher accuracy, is it finally determined that the metal object exists.

## 5. Discussion

In this paper, we experimented and analyzed the metal object detection method based on CNN and RNN using MI sensor. The CNN-based method images the signal acquired through the MI sensor. The imaged signal appears as a heatmap. By doing this, the noises are treated as the background and only the response of the signal can be learned. In this operation, it is difficult to extract features from an image when the sensor signal is weak. It is difficult to distinguish from signals in a static state, and accordingly, learning is difficult. The RNN-based method uses the signal acquired through the MI sensor as an input. RNN is advantageous for learning time-series data. This is because it learns interrelationships by judging the continuity of data. A high detection rate is shown for continuous data, but a low detection rate is shown for non-contiguous data. Since the data acquired from the MI sensor includes non-continuous data, we experimented with a learning method using CNN to compensate for the shortcomings of RNN. Although CNN predicted that a high detection rate would come out from non-contiguous data, it was difficult to judge the data due to the strength of the signal. The number of parameters between CNN and RNN is dramatically different. As a result, train cost and inference cost increase, increasing training and execution time. RNN showed high performance in terms of both temporal and accuracy.

In such an RNN, the raw data was not used directly, but rather as an input after noise removal. When noise is removed, the weak response of the signal is also removed or the continuity of the signal is ambiguous. In this respect, it was confirmed that the use of raw data without purification results in high performance.

## 6. Conclusions

In this study, we evaluated the CNN and RNN performances in detecting metal objects from measurement data from an AICHI AMI305-AR16 sensor using a deep learning network. In most deep learning networks, the deeper the layer is, the better the performance is. Networks that are too deep are difficult to learn and increase the amount of computation. This engenders limited performance in devices such as small computers and mobiles. To address this issue, the structures of the CNN and RNN networks with the depths of one, three, five, seven, and nine layers, and with the worst and best performances, were analyzed. The RNN showed a lower computational load and a higher performance than the CNN. In addition, using the fifth layer provided high performance for each distance between the object and the sensor. Future work may include development of a model optimized for sensor sensitivity over longer distances, rather than simply comparing and analyzing the model. In addition, a study on learning through GPU will be needed to construct a deep network to improve performance and to learn quickly.

## Figures and Tables

**Table 1:** 30 cm distance between the steel plate and sensor. Red is the best performance.

		1 Layer	3 Layers	5 Layers	7 Layers	9 Layers
**CNN**	**30 × 30 (cm)**	87.51%	89.10%	89.25%	88.30%	84.22%
	**50 × 50 (cm)**	86.85%	87.81%	85.81%	81.78%	84.33%
	**70 × 70 (cm)**	81.77%	84.72%	83.43%	89.45%	79.15%
	**Avg.Total**	85.37%	87.21%	86.16%	86.51%	82.56%
		**1 Layer**	**3 Layers**	**5 Layers**	**7 Layers**	**9 Layers**
**LSTM**	**30 × 30 (cm)**	91.04%	91.64%	95.67%	96.62%	90.20%
	**50 × 50 (cm)**	88.61%	91.73%	93.91%	94.42%	91.86%
	**70 × 70 (cm)**	90.99%	90.24%	97.28%	92.96%	93.98%
	**Avg.Total**	88.55%	91.20%	96.04%	94.66%	92.01%
		**1 Layer**	**3 Layers**	**5 Layers**	**7 Layers**	**9 Layers**
**GRU**	**30 × 30 (cm)**	93.47%	97.40%	95.72%	94.79%	91.33%
	**50 × 50 (cm)**	90.79%	95.31%	96.43%	88.35%	95.63%
	**70 × 70 (cm)**	90.01%	92.67%	90.06%	91.99%	94.63%
	**Avg.Total**	91.42%	95.12%	94.07%	90.76%	93.86%

**Table 2:** 40 cm distance between the steel plate and sensor. Red is the best performance.

		1 Layer	3 Layers	5 Layers	7 Layers	9 Layers
**CNN**	**30 × 30 (cm)**	81.57%	83.15%	84.27%	81.22%	85.77%
	**50 × 50 (cm)**	82.40%	85.12%	87.62%	85.12%	86.78%
	**70 × 70 (cm)**	87.51%	77.42%	87.13%	84.35%	81.57%
	**Avg.Total**	83.82%	81.89%	86.34%	83.56%	84.70%
		**1 Layer**	**3 Layers**	**5 Layers**	**7 Layers**	**9 Layers**
**LSTM**	**30 × 30 (cm)**	65.33%	80.23%	81.59%	77.70%	80.92%
	**50 × 50 (cm)**	91.61%	97.39%	97.98%	89.35%	96.70%
	**70 × 70 (cm)**	91.20%	93.72%	95.56%	94.66%	95.08%
	**Avg.Total**	82.71%	90.44%	91.71%	87.23%	90.90%
		**1 Layer**	**3 Layers**	**5 Layers**	**7 Layers**	**9 Layers**
**GRU**	**30 × 30 (cm)**	92.12%	92.32%	94.79%	97.06%	94.90%
	**50 × 50 (cm)**	92.10%	89.21%	89.78%	90.26%	89.44%
	**70 × 70 (cm)**	92.85%	86.76%	89.91%	91.22%	76.96%
	**Avg.Total**	92.35%	89.43%	91.49%	92.84%	87.10%

**Table 3:** 50 cm distance between the steel plate and sensor. Red is the best performance.

		1 Layer	3 Layers	5 Layers	7 Layers	9 Layers
**CNN**	**30 × 30 (cm)**	83.77%	81.44%	84.53%	85.72%	81.12%
	**50 × 50 (cm)**	82.11%	83.23%	80.57%	83.12%	82.15%
	**70 × 70 (cm)**	78.91%	77.15%	82.21%	80.75%	87.11%
	**Avg.Total**	81.59%	80.60%	82.43%	83.19%	83.46%
		**1 Layer**	**3 Layers**	**5 Layers**	**7 Layers**	**9 Layers**
**LSTM**	**30 × 30 (cm)**	57.97%	85.01%	84.55%	69.68%	83.80%
	**50 × 50 (cm)**	88.14%	89.48%	90.78%	94.66%	93.72%
	**70 × 70 (cm)**	91.97%	95.26%	95.02%	95.42%	70.67%
	**Avg.Total**	79.36%	89.91%	90.11%	86.58%	82.73%
		**1 Layer**	**3 Layers**	**5 Layers**	**7 Layers**	**9 Layers**
**GRU**	**30 × 30 (cm)**	91.13%	90.15%	88.19%	88.97%	85.27%
	**50 × 50 (cm)**	91.48%	91.51%	94.77%	95.45%	93.74%
	**70 × 70 (cm)**	71.57%	86.86%	89.65%	91.22%	84.10%
	**Avg.Total**	84.72%	89.50%	90.87%	91.88%	87.70%
